# m6A regulator-based methylation modification patterns and characterization of tumor microenvironment in acute myeloid leukemia

**DOI:** 10.3389/fgene.2022.948079

**Published:** 2022-08-10

**Authors:** Zi-Jun Xu, Xiang-Mei Wen, Yuan-Cui Zhang, Ye Jin, Ji-Chun Ma, Yu Gu, Xin-Yi Chen, Pei-Hui Xia, Wei Qian, Jiang Lin, Jun Qian

**Affiliations:** ^1^ Laboratory Center, Affiliated People’s Hospital of Jiangsu University, Zhenjiang, China; ^2^ Zhenjiang Clinical Research Center of Hematology, Affiliated People’s Hospital of Jiangsu University, Zhenjiang, China; ^3^ The Key Lab of Precision Diagnosis and Treatment in Hematologic Malignancies of Zhenjiang City, Affiliated People’s Hospital of Jiangsu University, Zhenjiang, China; ^4^ Department of Internal Medicine, The Affiliated Third Hospital of Jiangsu University, Zhenjiang, China; ^5^ Department of Hematology, Affiliated People’s Hospital of Jiangsu University, Zhenjiang, China; ^6^ Department of Otolaryngology-Head and Neck Surgery, Affiliated People’s Hospital of Jiangsu University, Zhenjiang, China

**Keywords:** m6A, acute myeloid leukemia, tumor microenvironment, immune infiltration, metabolic pathways

## Abstract

RNA N6-methyladenosine (m6A) is the most common and intensively studied RNA modification that critically regulates RNA metabolism, cell signaling, cell survival, and differentiation. However, the overall role of multiple m6A regulators in the tumor microenvironment (TME) has not yet been fully elucidated in acute myeloid leukemia (AML). In our study, we explored the genetic and transcriptional alterations of 23 m6A regulators in AML patients. Three distinct molecular subtypes were identified and associated with prognosis, patient clinicopathological features, as well as TME characteristics. The TME characterization revealed that m6A patterns were highly connected with metabolic pathways such as biosynthesis of unsaturated fatty acids, cysteine and methionine metabolism, and citrate cycle TCA cycle. Then, based on the differentially expressed genes (DEGs) related to m6A molecular subtypes, our study categorized the entire cohort into three m6A gene clusters. Furthermore, we constructed the m6Ascore for quantification of the m6A modification pattern of individual AML patients. It was found that the tumor-infiltrating lymphocyte cells (TILs) closely correlated with the three m6A clusters, three m6A gene clusters, and m6Ascore. And many biological processes were involved, including glycogen degradation, drug metabolism by cytochrome P450, pyruvate metabolism, and so on. Our comprehensive analysis of m6A regulators in AML demonstrated their potential roles in the clinicopathological features, prognosis, tumor microenvironment, and particularly metabolic pathways. These findings may improve our understanding of m6A regulators in AML and offer new perspectives on the assessment of prognosis and the development of anticancer strategy.

## Introduction

Acute myeloid leukemia (AML) is a highly fatal hematopoietic malignancy with excessive proliferation of immature leukemic blasts and poor prognosis (Dohner et al., 2017; Thomas and Majeti, 2017). The recurrence and mortality rates of AML are still very high with currently available therapeutics (Buckley et al., 2018; Koenig and Mims, 2020). The scientists have been dedicated to developing more novel and synergistic therapeutic targets of AML (Metzeler et al., 2016). A lot of work has been done on the role of gene transcription in leukemogenesis whereas the functional significance of posttranscriptional regulation of gene expression such as RNA modifications has received great attention from researchers in recent years.

Methylation of N6 adenosine (m6A), which has been discovered as reversible RNA methylation affecting the regulation of post-transcriptional gene expression programs and protein production, is one of the most abundant internal marks on mammalian mRNA (Desrosiers et al., 1974; Fu et al., 2014; Zhao et al., 2017; Shi et al., 2019). The biological functions altered by m6A modifications are dynamically controlled by the m6A methyltransferase complex (writers), m6A demethylases (erasers), and m6A-binding proteins (readers) (Shi et al., 2019; Zaccara et al., 2019). A deep understanding of these regulatory proteins is essential to our comprehension of the mechanisms of m6A in gene regulation. It has been reported that m6A modification is an important regulator of the development and regulation of normal and malignant hematopoiesis (Vu et al., 2019). Emerging evidence also demonstrates the involvement of m6A modification in physiological and pathological processes of hematopoiesis and leukemogenesis (Paris et al., 2019; Sheng et al., 2021). The m6A writers [methyltransferase-like 3 (METTL3), methyltransferase-like 14 (METTL14)], erasers [α-ketoglutarate-dependent dioxygenase AlkB homolog 5 (ALKBH5), and fat mass and obesity-associated protein (FTO)], highly expressed in AML, were involved in the development, differentiation, progression, and maintenance of AML through various m6A-dependent mechanisms (Barbieri et al., 2017; Li et al., 2017; Vu et al., 2017; Weng et al., 2018; Shen et al., 2020). m6A reader gene YT521-B homology (YTH) domain family protein 2 (YTHDF2) is also upregulated and plays an important role in tumor promotion in AML development/maintenance (Paris et al., 2019).

The tumor microenvironment (TME), a complex ecosystem that includes malignant and non-malignant cells, immune cells, stromal cells, and other components, plays a crucial role in tumor initiation and progression (Friedl and Alexander, 2011; Hanahan and Weinberg, 2011; Binnewies et al., 2018). Accommodation of tumor cells with their nearby milieu promotes to evolve hallmarks related to the tumorigenesis, which is affected from cellular differentiation state of immune and stromal cells, normalizing this milieu will explore paths to therapy (Mortezaee, 2021; Mortezaee and Majidpoor, 2021; Mortezaee and Majidpoor, 2022). Also, cancer cells autonomously alter their metabolic pathways since they need abundant energy and raw materials required for proliferation and survival (Hanahan and Weinberg, 2011; Li and Zhang, 2016). The researchers show that the modification of m6A is also implicated in the regulation of physiology and metabolism in tumors (Faubert et al., 2017; Choe et al., 2018). Leukemia-associated alterations within the AML niche such as increased hypoxia, inflammation and metabolic reprogramming facilitate immune evasion and chemotherapy resistance as well as contribute to AML progression (Mendez-Ferrer et al., 2020). Liu et al. manifested that the m6A demethylase FTO could regulate glycolytic metabolism which induced tumors to escape immune surveillance (Liu et al., 2021). Nevertheless, most studies focus on only individual or small numbers of m6A regulators due to technical limitations. How multiple m6A regulators mediate the micro-environmental phenotype which may provide important insights for understanding the underlying mechanism of AML tumorigenesis is not well elaborated.

In this study, we obtained the datasets of AML from the Cancer Genome Atlas (TCGA). Next, we constructed the three m6A clusters, three m6A gene clusters, and a set of scoring system step by step and comprehensively evaluated the association of m6A modification patterns with clinical outcomes, biological pathways, and TME characteristics. All in all, we attempted to uncover the mechanism of m6A modification patterns in the AML development, and subsequently provided references for future research and a basis for clinical treatment.

## Materials and methods

### Data acquisition

**FIGURE 1:**
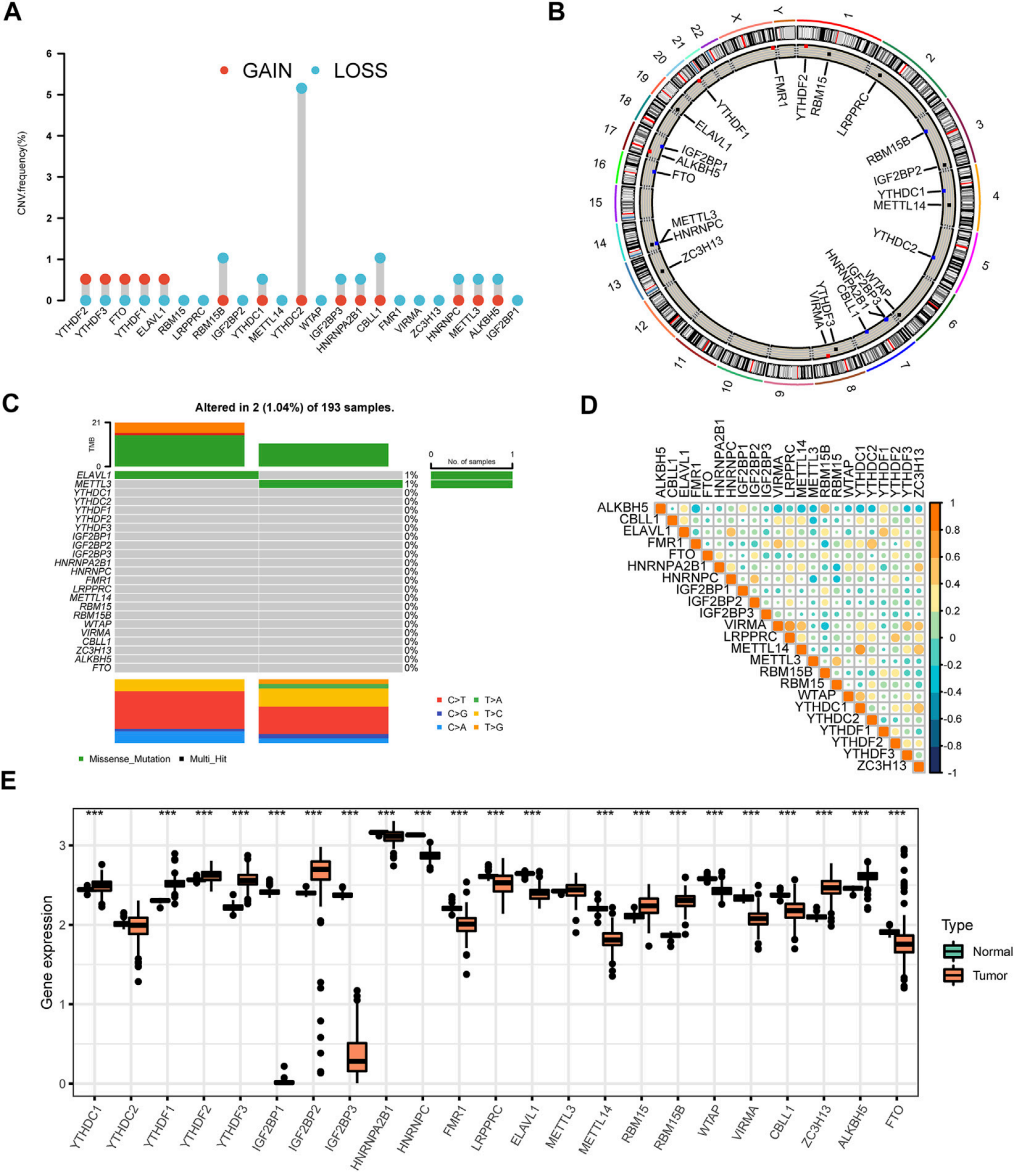
The panorama of genetic variations and expression patterns of m6A regulators in AML **(A)** The CNV variation frequency of 23 m6A regulators was prevalent from the TCGA cohort. The height of the column denoted the alteration frequency. The amplification frequency was labeled as a red dot. The deletion frequency was labeled as a blue dot **(B)** The location of CNV alteration of m6A regulators on chromosomes. The black dot, the red dot, and the blue dot in the ring indicated no copy number change, amplification, and deletion respectively **(C)** The genetic alteration frequency of 23 m6A regulators in 193 patients with AML. The upper bar plot showed TMB. The panel on the right represented the mutation frequency and proportion of each variant type in each regulator **(D)** Correlation of the investigated m6A modification regulators **(E)** The differences of expression levels of 23 m6A regulators between normal and AML samples (***p < 0.001).

The multi-omics data of AML analyzed in Figure 1, including copy number variation (CNV), single nucleotide variation (SNV), and mRNA (RNAseq) data, were all downloaded from the GDC of the TCGA portal (https://portal.gdc.cancer.gov/repository). This TCGA AML data include bulk specimen from diagnosed AML patients. Patients without survival information were excluded and a total of 151 AML patients were included for further evaluation. In Supplementary Figure S1B, we analyzed gene expression data from normal cell populations, which was part of the Hemap data retrieved from a previous publication (Dufva et al., 2020). These were specimens of sorted immune cells. For Supplementary Figure S1C, we assessed the expression of 23 m6A regulators in bulk gene expression data from pan-hematological malignancies, which was also part of the Hemap data. We also used a published scRNA-seq data from 16 AML samples at diagnosis consisting of 30,712 BM cells (Van Galen AML scRNA), which were downloaded from GEO with accession GSE116256.

### Consensus molecular clustering for 23 m6A regulators

A total of 23 acknowledged m6A regulators were analyzed for identifying different m6A modification patterns. These 23 m6A regulators comprised two erasers (ALKBH5, FTO), 13 readers (YTHDC1, YTHDC2, YTHDF1, YTHDF2, YTHDF3, IGF2BP1, IGF2BP2, IGF2BP3, HNRNPA2B1, HNRNPC, FMR1, LRPPRC, ELAVL1), and 8 writers (METTL3, METTL14, RBM15, RBM15B, WTAP, VIRMA, CBLL1, ZC3H13). We applied the R package “ConsensusClusterPlus” to classify patients from the TCGA AML cohort into distinct m6A modification patterns according to the expression of 23 m6A regulators. Next, we compared the relationships between m6A molecular subtypes and clinicopathological characteristics including FAB subtypes, cytogenetic risk, gender, age, and WBC.

### Differentially expressed genes identification

m6A modification-related DEGs were checked among different m6A subtypes using the empirical Bayesian approach of the limma R package. We categorized the cases into different subtypes (gene cluster A, gene cluster B, and gene cluster C) for further analysis. In practice, we used the voom algorithm to normalize gene expression data and then input lmFit and eBayes functions to calculate the statistics for differential expression with a corrected *p*-value < 0.05. GO annotation for m6A phenotype-related genes was performed in the R package “clusterProfiler” with the cutoff value of FDR <0.05. Kaplan-Meier curves analysis was employed to measure the discrepancy of overall survival (OS) between different gene clusters.

### Generation of m6A gene signature

The m6Ascore was set up to assess the m6A modification patterns of individual patients with AML by using principal component analysis (PCA). To be more specific, a total of 70 overlap DEGs were subjected to univariate Cox regression analysis to identify a set of candidate prognostic genes. And we screened out 25 genes with a *p* value less than 0.05 by the random forest-recursive feature elimination (RFE) method with 10-fold cross-validation in the R package “caret”. Then we conducted the PCA analysis based on the expression profiles of the final determined genes. Principal components 1 and 2 were both selected as signature scores. As a result, the m6Ascore was calculated as follows: m6Ascore = Σ(PC1i + PC2i), where i was the expression of m6A phenotype-related genes (Zeng et al., 2019). m6Ascore was the integral of a continuous variable and the m6Ascore group was a binary variable obtained according to the X tile method.

### Gene set variation analysis and TME cell infiltration estimation

We employed GSVA enrichment analysis to evaluate the disparity involved in physiological processes and biological functions among different m6A modification patterns by using “GSVA” R packages. Additionally, we adopted the CIBERSORT algorithm to calculate the relative abundance of 23 tumor-infiltrating lymphocyte cells (TILs) in different m6A modification patterns with the gene expression profile (Newman et al., 2015). And the 23 cell types included B cells, T cells, dendritic cells, natural killer cells, eosinophil, macrophages, monocyte, neutrophil, and myeloid subsets. A violin map visualized the difference of immune cell abundance among m6A modification patterns. To extract detailed mutational information, Mutation Annotation Format data files based on TCGA cohort were depicted by the waterfall function of the R “maftools” package.

### Statistical analyses

We generated statistical analyses in this study with R software version 4.0.4 (https://www.r-project.org/). For comparison of two continuous variables, statistical significance was evaluated by the Chi-square test and Fisher’s exact test. Univariate Cox regression and Kaplan-Meier curves were used to test the prognostic effects of the different subtypes with the “survival” and “survminer” R packages. Samples were allocated into high and low m6Ascore subgroups with the surv-cutpoint function from the “survminer” package. The two-sided *p* < 0.05 in all comparisons was considered to indicate a statistically significant difference.

## Results

### Genetic and transcriptional alterations of m6A regulators in AML

We investigated a total of 23 m6A regulators including 8 writers, two erasers, and 13 readers in this study (Supplementary Table S1). The differences in CNV of all regulators were explored. The CNV of m6A regulators was prevalent in AML. The CNV was found to be focused on the deletion predominantly, while YTHDF2, YTHDF3, FTO, YTHDF1, and ELAVL1 had more prevalent amplification frequency (Figure 1A,Supplementary Table S2). The locations of CNV alteration in 23 m6A regulators on their respective chromosomes were displayed in Figure 1B (Supplementary Tables S3, S4). Moreover, we compared AML samples to normal samples using PCA analysis based on paired tumor-normal specimens and found that AML samples were clearly distinguished from normal samples by the 23 m6A regulators (Supplementary Figure S1A). After evaluating the incidence of somatic mutations of 23 m6A regulators in AML, we concluded that the alteration frequency was only 1.04% among 193 samples with two missense mutations identified in METTL3 and RBMX (Figure 1C). Notably, m6A regulators are widely known for their collaboration in cancer progression. The relevance of the co-expression of regulators was therefore analyzed, and LRPPRC and VIRMA owned a significant correlation with the highest correlation coefficient (R = 0.66) (Figure 1D, Supplementary Tables S5, S6). Intrigued by these findings, we further compared the mRNA expression levels between AML and normal tissues. The results displayed all of the 23 m6A regulators showed significant differences between AML and normal tissues except for YTHDF2 and METTL3 (Figure 1E). Beyond that, m6A highlighted distinct patterns in different immune cell types, myeloid malignancies along with lymphoid malignancies (Supplementary Figure S1B,C). The observed clustering suggested that the cell-of-origin and different disease groups influenced the repertoire of m6A regulators. YTHDF3 presented relatively higher expression compared with the other 22 m6A regulators in M2-macrophage. At the same time, AML highly expressed IGF2BP2.

### Identification of m6A subtypes in AML

**FIGURE 2:**
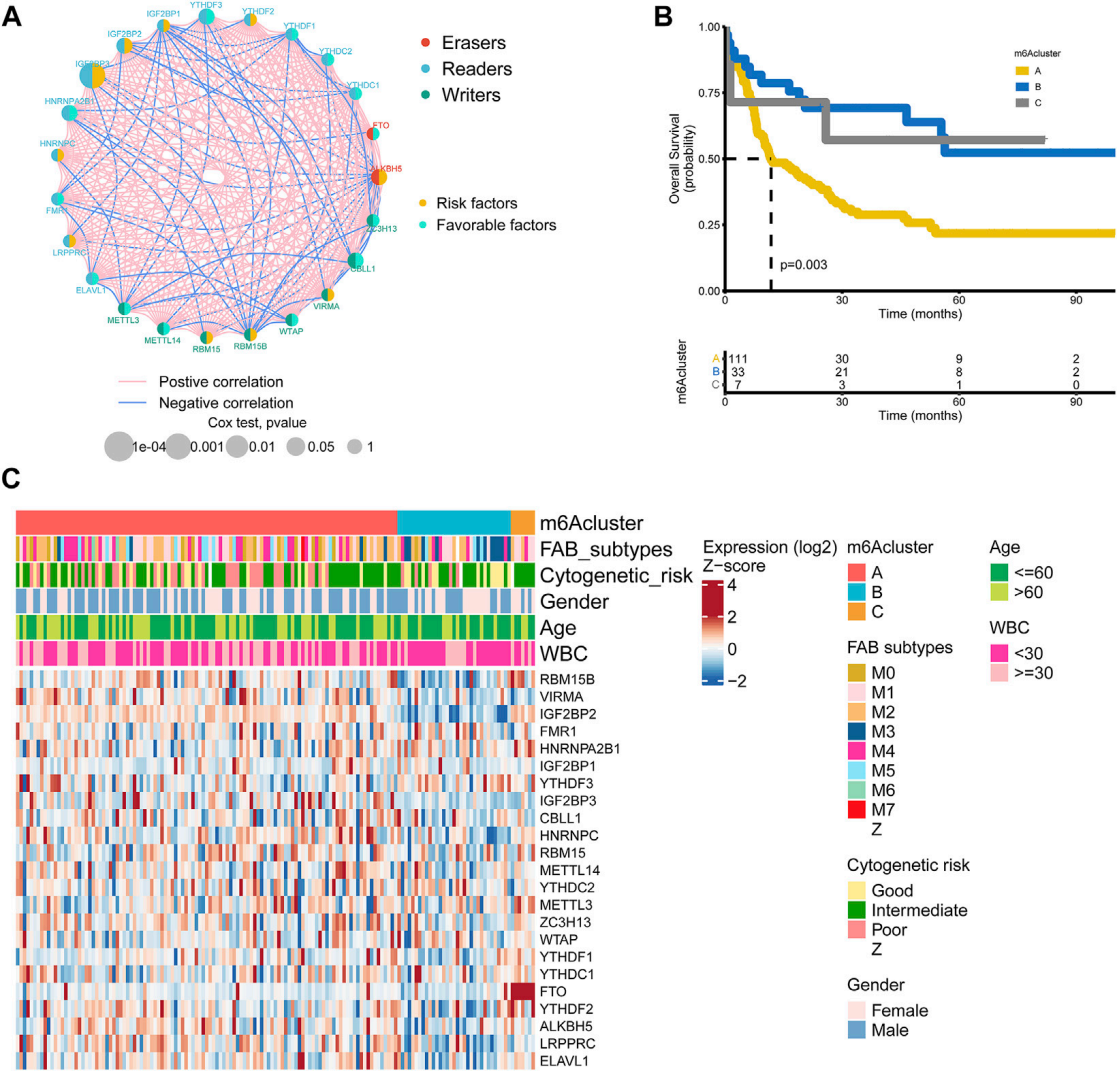
m6A regulators subtypes and their clinicopathological characteristics in AML **(A)** The interaction among m6A regulators in AML. The lines represented their interactions between regulators, and the thickness of lines showed the strength of the correlation. Effects of regulators on the prognosis were represented with the circle size, and *p*-values were calculated by Log-rank test (*p* < 1, *p* < 0.05, *p* < 0.01, *p* < 0.001, *P* < 1e-04) **(B)** Kaplan-Meier overall survival curves of 111 cases in m6A cluster A, 33 cases in m6A cluster B, and 7 cases in m6A cluster C from the TCGA AML cohort **(C)** Differences in clinicopathological characteristics and expression levels of m6A among the three distinct m6A clusters. The m6A cluster, FAB subtypes, cytogenetic risk, gender, age, and WBC were used as patient annotations.

151 patients from the TCGA dataset with available OS data and clinical information were enrolled in our study for further analysis (Supplementary Table S7). The results of univariate Cox regression analysis revealed the prognostic values of 23 m6A regulators in patients with AML (Supplementary Table S8). Figure 2A showed the comprehensive landscapes of 23 m6A regulators concerning their interactions, connections, and prognostic values in AML in the form of a m6A regulator network. Significant correlations existed among writers, erasers, and readers. We further explored the expression pattern of m6A regulators in AML and conducted the R package of ConsensusClusterPlus to classify the patients based on the expression level of 23 m6A regulators. It was clear that the clustering model was optimal when K = 3 during the process of K ranging from2 to 9 (Supplementary Figures S2A–S2C). The entire TCGA AML cohort was eventually allocated into three distinct modification patterns consisting of 111 cases in cluster A, 33 cases in cluster B, and 7 cases in cluster C (Figure 2B). The Kaplan-Meier curves for the three main m6A modification subtypes revealed the worst overall survival in patients with m6Acluster A (*p* = 0.003, Figure 2B). The prognosis of m6Acluster-B and m6Acluster C was similar in the Kaplan-Meier analysis, so we combined m6Acluster B with m6Acluster C and m6Acluster A was compared with it using univariate analyses. m6Acluster A had significantly poorer prognosis (HR = 0.3998, *p* < 0.01, Supplementary Table S9). Shen et al. have also reported m6A-based subtypes were remarkably related with overall survival in pan-cancer including AML (Shen et al., 2021). Moreover, we validated the biological characteristics of three m6A subtypes. The m6Acluster B was preferentially related to FAB subtypes M3, and the m6Acluster C subtype was markedly correlated with intermediate cytogenetic risk and FTO (Figure 2C). It was reported that FTO played a critical oncogenic role in AML (Li et al., 2017). We also observed differential expression patterns of m6A regulators among three clusters. For example, VIRMA, which plays an oncogenic role in multiple human cancers (Zhu et al., 2021), was more highly expressed in m6Acluster A compared with the other two clusters. Another gene with relatively higher expression in m6Acluster A-IGF2BP3-was recently found to be specifically overexpressed in AML and contributes to tumorigenesis and poor prognosis of this disease (Zhang et al., 2022). These findings agreed favorably with the observed negative prognostic influence of m6Acluster A.

### Associations of TME with three m6A subtypes

**FIGURE 3:**
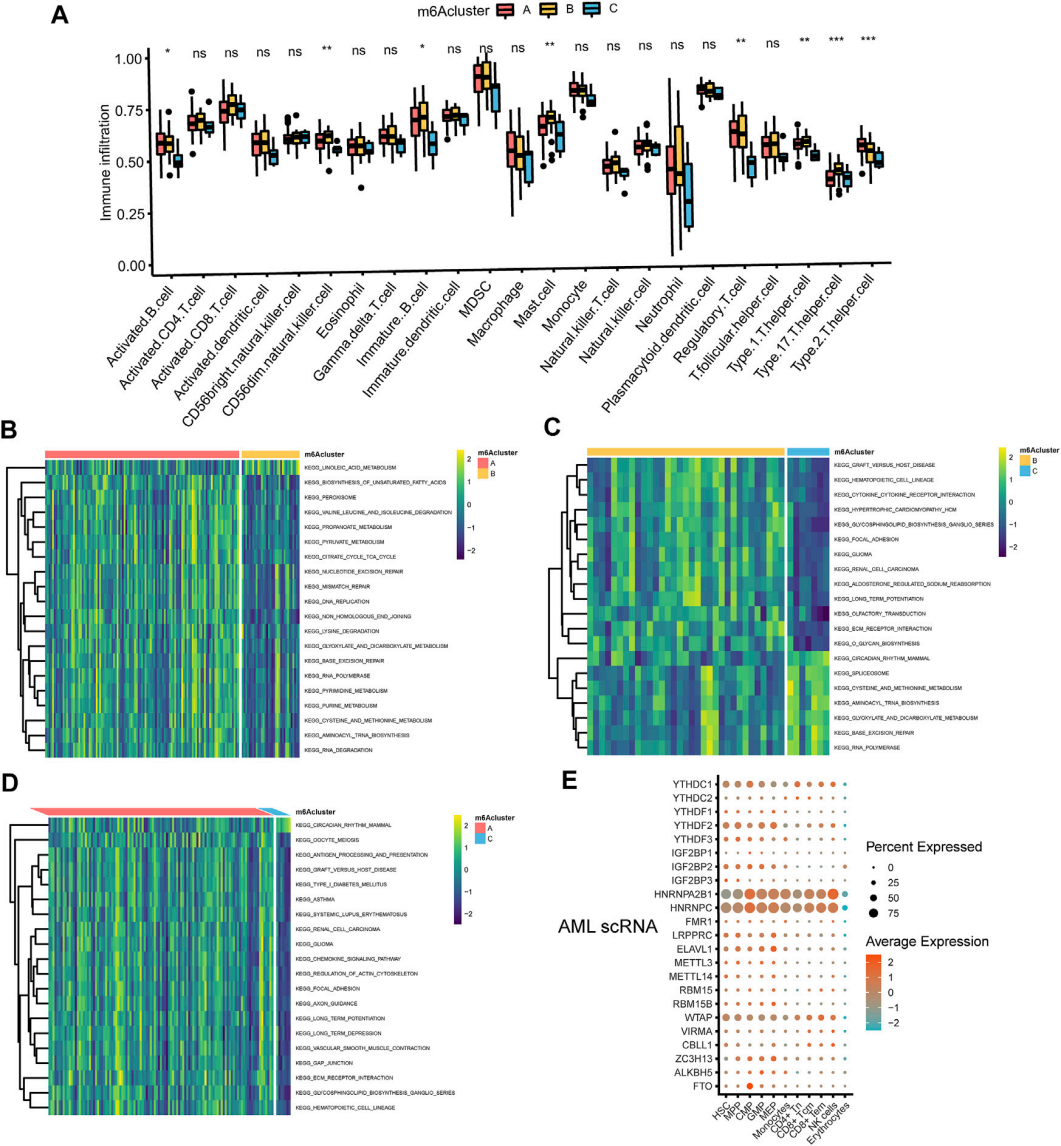
The interaction between TME and three m6A subtypes **(A)** The abundance of tumor-infiltrating lymphocyte cells in three m6A clusters. Violin plots displayed the differences in the immune cell distribution across three distinct subtypes. *p < 0.05, **p < 0.01, ***p < 0.01, ns, not significant **(B–D)** GSVA enrichment analysis of biological pathways in three distinct subtypes, visualized by the heatmap. B, m6Acluster A vs. m6Acluster B; C, m6Acluster B vs. m6Acluster C; D, m6Acluster A vs. m6Acluster C. **(E)** Dot plot of cell type assignment for AML scRNA expressed genes. Dot plot charts of the average expression were shown in different colors, and the percentage of cells with detectable expression was demonstrated in the size of the dot.

The association of 23 TILs with three m6A clusters was investigated by using the CIBERSORT algorithm in the TME of AML. We observed profound differences in immune cell infiltration including activated B cell, CD56dim natural killer cell, immature B cell, mast cell, regulatory T cell, type 1 T helper cell, type 17 T helper cell, and type 2 T helper cell among the three subtypes (Figure 3A). In addition, we performed GSVA enrichment analysis to detect the biological characteristics of three distinct m6A modification patterns. GSVA enrichment analysis showed that m6Acluster A was significantly enriched in metabolic pathways such as biosynthesis of unsaturated fatty acids, propanoate metabolism, pyruvate metabolism, citrate cycle TCA cycle, glyoxylate and dicarboxylate metabolism, pyrimidine metabolism, purine metabolism, cysteine and methionine metabolism (Figure 3B and Supplementary Table S10). m6Acluster B presented enrichment pathways highly related to metabolic pathways including linoleic acid metabolism (Figure 3B and Supplementary Table S10), and carcinogenic activation pathways such as ECM receptor interaction (Figure 3C and Supplementary Table S11). Likewise, m6Acluster C was prominently associated with metabolic pathways which encompassed cysteine and methionine metabolism, glyoxylate and dicarboxylate metabolism (Figure 3C and Supplementary Table S11). What’s more, m6Acluster A correlated with immune pathways and immune-related diseases simultaneously, including antigen processing and presentation, chemokine signaling pathway activation, asthma, and systemic lupus erythematosus (Figure 3D and Supplementary Table S12). Figure 3E illustrated cell type assignment for the scRNA expressed genes in AML.

### m6A phenotype-related DEGs in AML

**FIGURE 4:**
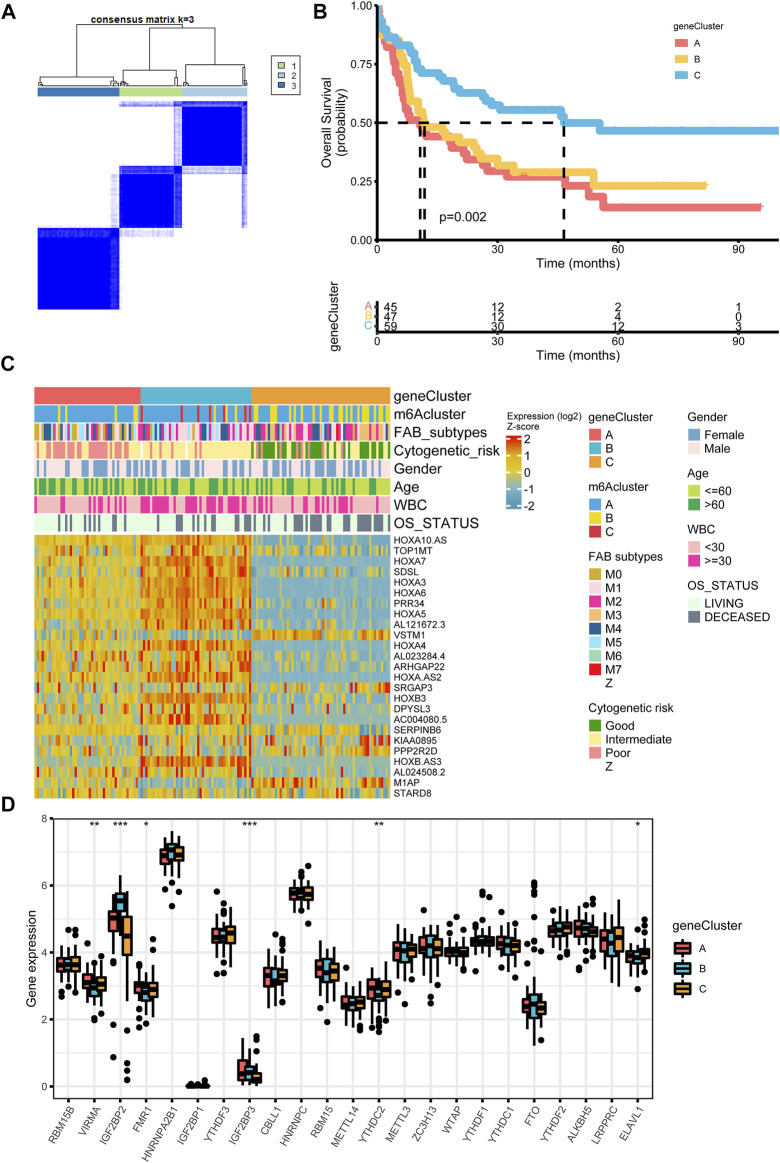
Construction of differential expression of m6A gene signatures **(A)** Unsupervised clustering of m6A phenotype-related genes in the TCGA cohort and consensus matrices for k = 3 **(B)** Kaplan-Meier curves for overall survival of the m6A phenotype-related gene signatures (*p* = 0.002, Log-rank test) **(C)** Relationships between clinicopathologic features and the three genomic subtypes. The gene clusters, m6A clusters, FAB subtypes, cytogenetic risk, gender, age, white blood cell, and overall survival status were used as patient annotations **(D)** Differences in the expression of 23 m6A regulators in the three gene clusters. *p < 0.05; **p < 0.01; ***p < 0.001.

To further investigate the underlying m6A-related transcriptional expression differences within three m6A modification patterns, we did some further analysis. Actually, we applied a Venn diagram to illustrate the DEGs among the three m6A modification patterns and 70 m6A phenotype-related overlap genes were presented (Supplementary Figure S3A, Supplementary Tables S13–S16). To further elaborate on the function of DEGs, we carried out a GO enrichment analysis. The result elucidated that enrichment of the biological processes related to embryonic organ morphogenesis/development (Supplementary Figure S3B). Supplementary Table S17 showed the expression level of 70 overlap DEGs. Univariate Cox regression study of 70 genes was analyzed and 25 genes with *p*-value less than 0.05 were screened out (Supplementary Table S18). Then we performed an unsupervised clustering algorithm to classify the entire cohort into three m6A gene signature subtypes, named as m6A gene cluster A-C, respectively (Figure 4A). Further survival analysis indicated three m6A genomic phenotypes showed significant prognostic differences in AML samples, of which 45 cases were in gene cluster A, 47 cases in gene cluster B, and 59 cases in gene cluster C (*p* = 0.002, Log-rank test, Figure 4B). Patients in gene cluster C were proved to be related to a better prognosis, while patients in gene cluster A and gene cluster B were associated with the outcome of poorer prognosis. We also observed that m6A gene cluster C patterns were associated with good cytogenetic risk, while m6A gene cluster A presented poor cytogenetic risk and m6A gene cluster B showed intermediate cytogenetic risk. Patients with alive status were largely concentrated in the m6A gene cluster A patterns. Interestingly, VSTM1 showed its highest expression level in the m6A gene cluster C, whereas M1AP was expressed lowest in the m6A gene cluster B (Figure 4C). High expression of VSTM1 was reported to be associated with a more favorable clinical outcome (Lai et al., 2021). The three m6A gene clusters indicated significant differences in the expression of VIRMA, IGF2BP2, FMR1, IGF2BP3, ELAVL1, and YTHDC2 (Figure 4D).

**FIGURE 5:**
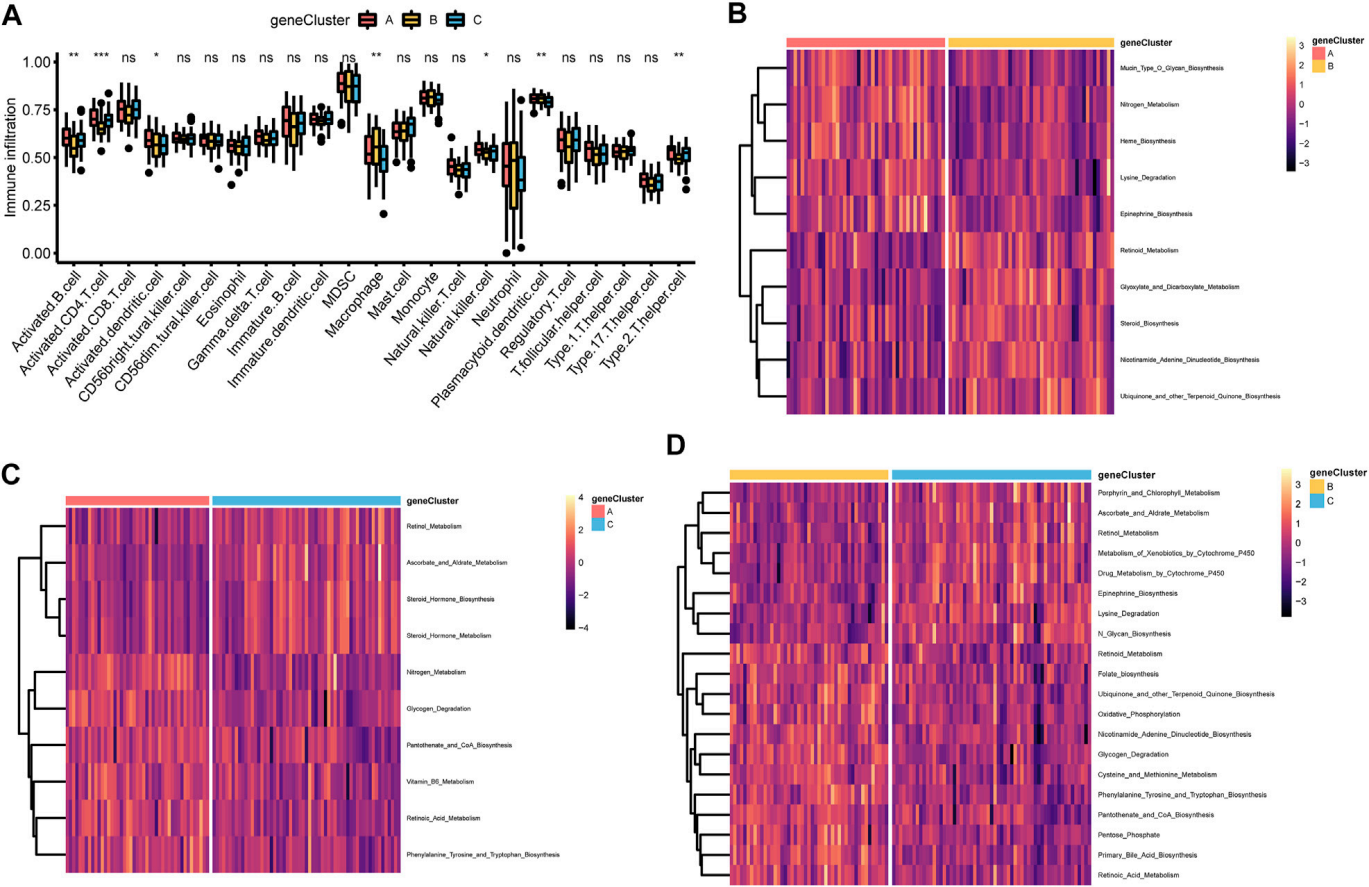
Correlations of TME and three m6A gene clusters **(A)** The fraction of tumor-infiltrating lymphocyte cells in three m6A gene clusters using the CIBERSORT algorithm. Violin plots illustrated the differences in the immune cell distribution across three m6A gene clusters. *p < 0.05, **p < 0.01, ***p < 0.01, ns, not significant **(B–D)** Heatmap was plotted to show the activation states of biological pathways in three m6A gene clusters via the GSVA enrichment analysis. B, m6A gene cluster A vs. m6A gene cluster B; C, m6A gene cluster B vs. m6A gene cluster C; D, m6A gene cluster A vs. m6A gene cluster C.

In addition, the immune cell infiltration of activated B cell, activated CD4 T cell, activated dendritic cell, macrophage, NK cell, plasmacytoid dendritic cell and type 2 T helper cell showed statistically significant differences across the three m6A gene clusters (Figure 5A). Meanwhile, m6A gene cluster A was prominently associated with metabolic pathways or biological process including mucin type O glycan biosynthesis, nitrogen metabolism, heme biosynthesis, lysine degradation, epinephrine biosynthesis, glycogen degradation, pantothenate and CoA biosynthesis, vitamin B6 metabolism, retinoic acid metabolism, phenylalanine tyrosine and tryptophan biosynthesis (Figure 5B, Figure 5C and Supplementary Table S19, Supplementary Table S20). m6A gene cluster B was associated with retinoid metabolism, glyoxylate and dicarboxylate metabolism, steroid biosynthesis, nicotinamide adenine dinucleotide biosynthesis, ubiquinone, and other terpenoid quinone biosynthesis, folate biosynthesis, oxidative phosphorylation, glycogen degradation, cysteine and methionine metabolism, phenylalanine tyrosine and tryptophan biosynthesis, pantothenate and CoA biosynthesis, pentose phosphate, primary bile acid biosynthesis, and retinoic acid metabolism (Figure 5C, Figure 5D, and Supplementary Table S20, Supplementary Table S21). m6A gene cluster C presented enrichment pathways prominently associated with retinol metabolism, ascorbate and aldrate metabolism, steroid hormone biosynthesis and metabolism, porphyrin and chlorophyll metabolism, metabolism of xenobiotics by cytochrome P450, drug metabolism by cytochrome P450, epinephrine biosynthesis, lysine degradation, and N glycan biosynthesis (Figure 5C, Figure 5D, and Supplementary Tables S20, S21).

### Construction of m6A score and the relevance of clinical features

**FIGURE 6:**
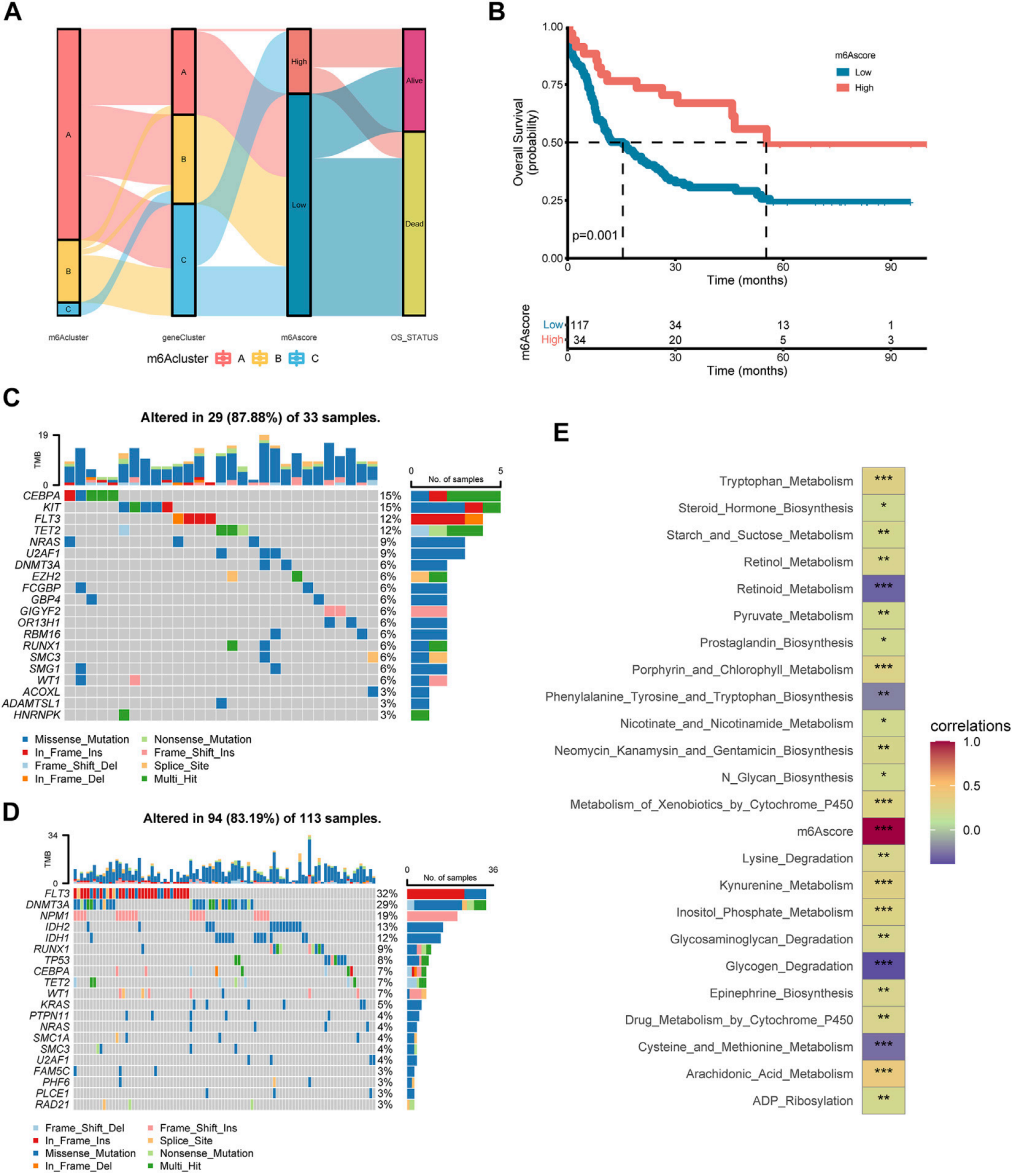
Construction of the m6A score in the training set and the relevance of clinical features **(A)** Alluvial diagram of subtype distributions in groups with different m6A clusters, m6A gene clusters, m6A score, and OS status **(B)** Kaplan-Meier analysis for high m6A score and low m6A score groups in AML cohort (*p* = 0.001, Log-rank test) **(C–D)** Oncoplot visualized the frequently mutated genes in the high m6Ascore group **(C)** and the low m6Ascore group **(D)**. In the above two figures, the top 20 mutated genes were distributed vertically by reducing mutation frequency from top to bottom **(E)** Functional annotation for m6Ascore using GSVA enrichment analysis. The intensity of the colors represents the strength of correlation.

Considering the individual heterogeneity and complexity of m6A modification, a scoring system based on the signature genes related to m6A, named m6Ascore, was established to quantify the m6A modification pattern of single AML patient (Supplementary Table S22). Figure 6A illustrated an alluvial diagram of three m6A clusters, three m6A gene clusters, m6Ascore, and OS status. Remarkably, m6A cluster B exhibited the highest m6A score, followed by m6A cluster A and m6A cluster C (Supplementary Figure S4A). The results suggested that the m6Ascore of m6A gene cluster B was the lowest, while that of m6A gene cluster C was the highest (Supplementary Figure S4B). Spearman analysis was applied to better understand the correlation between immune cells and the m6Ascore (Supplementary Figure S4C and Supplementary Table S23). m6Ascore was found to be positively associated with regulatory T cell. To further shed light on the impact of m6Ascore in AML, we next explored its potential value for predicting clinical outcomes. Overall survival curves of Kaplan-Meier analyses revealed that patients with high m6Ascore had a significantly prominent prognosis (*p* = 0.001, Log-rank test, Figure 6B). Similarly, by univariate analyses of the TCGA data set, the high m6Ascore was significantly related to better OS (HR = 2.442, *p* < 0.01, Supplementary Table S24). To explore the distribution variations of the somatic mutations between low and high m6Ascore, we evaluated samples from the TCGA dataset using the maftools package. The top five mutated genes in the high m6Ascore group were *CEBPA*, *KIT*, *FLT3*, *TET2*, and *NRAS*, in parallel, the top five mutated genes in the low m6Ascore group were *FLT3*, *DNMT3A*, *NPM1*, *IDH2*, and *IDH1* (Figures 6C,D). The Forest plot revealed the alteration frequencies of the top five mutated genes in two groups using the chi-square test (Supplementary Figure S4D). Delightfully, we found m6Ascore correlated strongly with tryptophan metabolism, pyruvate metabolism, glycogen degradation, cysteine and methionine metabolism, arachidonic acid metabolism, and ADP ribosylation (Figure 6E). There has been a study indicating that AML-related metabolic differences involved in tryptophan metabolism (Wang D. et al., 2019).

## Discussion

As one of the most common, abundant, and intensively studied RNA modifications. m6A has been regarded as an important epigenetic mark that critically regulates RNA metabolism, cell signaling, cell survival, and differentiation which impacts the behavior and phenotype of cells (Meyer and Jaffrey, 2017; Roundtree et al., 2017). A recent wave of studies has provided critical insights that m6A modifications play an important part in leukemia (Elkashef et al., 2017; Vu et al., 2017; Weng et al., 2018; Shen et al., 2020). Nevertheless, most studies focus on the single regulator, and the overall role of multiple m6A regulators has not yet been fully elucidated. We aimed to characterize the association of integrated m6A modification patterns with TME which would offer us a deeper and richer understanding in the prognosis and development of AML.

Our study discovered that the relative abundance of 23 TILs closely correlated with the three m6A clusters, three m6A gene clusters, and m6Ascore. Nowadays, m6A modification has been found to play a critical role in tuning the immune response (Shulman and Stern-Ginossar, 2020). It is demonstrated that YTHDF1 is essential for durable neoantigen-specific immunity, and YTHDF1-deficient mices show an elevated antitumor response of tumor-infiltrating CD8^+^ T cell (Han et al., 2019). METTL3-mediated mRNA m6A modification participates in the physiological promotion of dendritic cell activation (Wang H. et al., 2019). Indeed, leukemic blasts abnormally express the ligands for Immune Checkpoints (ICs) to escape immune supervision and present a series of metabolic alterations that could be involved in immunoregulation (Mougiakakos, 2019; Toffalori et al., 2019).

The present study recognized evident features in 23 m6A regulators at the transcriptional and genetic levels in AML. We also constructed three distinct m6A methylation modification patterns based on 23 m6A regulators. Compared to patients with m6A cluster B and m6A cluster C, m6A cluster A group had a worse outcome. The characteristics of the GSVA enrichment analysis also differed significantly between the three subtypes. Our data showed that m6Acluster A was significantly enriched in metabolic pathways such as biosynthesis of unsaturated fatty acids, cysteine and methionine metabolism, and citrate cycle TCA cycle. It has been reported that METTL3 upregulates fatty acid synthase which reduces hepatic insulin sensitivity (Xie et al., 2019). Fatty acid metabolism disorders participated in AML-induced metabolic reprogramming (Wang D. et al., 2019). Additionally, METTL3 enhances lipid accumulation via downregulating peroxisome proliferator-activator *α* (PPARα) expression (Zhong et al., 2018). In previous studies, methionine metabolism was reported to be strongly related to m6A methylation (Cao et al., 2016). And the free methionine can be converted to S-adenosylmethionine (SAM) (Anstee and Day, 2012). Homeostatic regulation of SAM synthesis encompasses dynamic m6A modifications in the MAT2A 3′ UTR of mammalian cells (Shima et al., 2017). In recent years, the TCA cycle has also re-emerged as a pivotal metabolic hub that supports tumor growth in both mouse models of cancer and patients with cancer (Martinez-Reyes et al., 2020; Krall et al., 2021).

Further, we identified three gene subtypes based on the DEGs between the three m6A clusters. The results concluded gene clusters could serve as valuable indexes for evaluating prognosis. In addition, m6A gene cluster A was prominently associated with biological process including glycogen degradation, and m6A gene cluster C was prominently associated with drug metabolism by cytochrome P450. Scholars have reported that R-2-hydroxyglutarate (R-2HG) abates FTO/m6A/YTHDF2-mediated upregulation of phosphofructokinase platelet (PFKP) and lactate dehydrogenase B (LDHB) which suppress aerobic glycolysis and exert an anti-tumor effect in R-2HG-sensitive leukemia cells (Qing et al., 2021). Likewise, targeting FTO/MYC/CEBPA signaling, R-2HG displays an intrinsic and broad anti-tumor activity in leukemia (Su et al., 2018). On the same lines, it is demonstrated that m6A modification regulates the expression level of drug-metabolizing enzymes P450 (Nakano et al., 2020; Nakano and Nakajima, 2022). And m6A modification affects drug and lipid metabolism by regulating the regulation of CES2 (Takemoto et al., 2021). All of this verified again that the DEGs were deemed to be m6A phenotype-related gene signatures and the m6A modification played an important role in shaping different TME landscapes.

We next established a quantification system to define different m6A modification patterns of individual patients. Patients with low- and high-risk m6Ascores exhibited significantly different clinicopathological characteristics, prognosis, and mutation, respectively. What’s more, m6Ascore correlated strongly with tryptophan metabolism, pyruvate metabolism, nicotinate, and nicotinamide metabolism, glycogen degradation, cysteine and methionine metabolism, arachidonic acid metabolism, ADP ribosylation, et al. Monocarboxylates such as lactate, pyruvate, and the ketone bodies play essential roles in metabolism (Halestrap, 2013). As reported in the literature, pyruvate can be transformed into lactate *via* lactate dehydrogenase (LDH) which is frequently upregulated in multiple cancers (Wang et al., 2012). Moreover, pyruvate carboxylase (PC) which is essential for primary and metastatic tumor growth catalyzes pyruvate to the TCA cycle metabolite oxaloacetate (Christen et al., 2016). Wang et al. tested nicotinamide adenine dinucleotide phosphate (NADP) binds to FTO, which led to decreased m6A methylation in RNA and adipogenesis (Wang et al., 2020). Altered metabolic program has been mainly described in solid tumors, and has recently gained attention in hematological cancers, including AML. It was found that AML cells presenting FLT3-ITD mutations have enhanced glycolytic activity, primarily due to higher phosphorylation of HK2 localized to mitochondria, favoring ATP transfer from OxPHOS to glycolysis (Ju et al., 2017). It was also shown that a combination of glycolytic inhibitors with FLT3-ITD inhibitors produced encouraging results *in vivo* (Huang et al., 2016; Ju et al., 2017). These findings showed an active involvement of metabolic program in AML and the promise of targeting this process.

In this study, our comprehensive analysis based on 23 m6A regulators suggested the association of the m6A modification patterns with TME, clinicopathological features, and prognosis in AML. Moreover, various metabolic mechanisms related to the m6A’s modification were discovered. Given metabolic reprogramming of tumor cells plays an extremely important role in tumor initiation and progression, focusing on m6A modification with altered metabolic pathways may be a promising anticancer strategy. In the same manner, elucidation of the significance of m6A in the regulation of the TME is expected to lead to a deeper understanding of the occurrence and development of AML.

## Data Availability

The original contributions presented in the study are included in the article/Supplementary Material, further inquiries can be directed to the corresponding authors.

## Supplementary material

Supplementary Figure 1 Characterization analysis of the 23 m6A regulators. **(A)** Principal component analysis 23 m6A regulators based on paired tumor-normal specimens. **(B, C)** Expression heat map of 23 m6A regulators in different types of immune cells **(B)** and hematological malignancies **(C)**.

Supplementary Figure 2 Unsupervised clustering of 23 m6A regulators in the TCGA cohort. **(A, B)**Consensus clustering analysis presented the relative change in the area under the cumulative distribution function (CDF) when k = 2−9. **(C)** The samples were clustered into cluster1, cluster2, and cluster3 when k = 3.

Supplementary Figure 3 m6A phenotype-related DEGs in AML. **(A)** Venn diagram of 70 m6A-related DEGs among three m6A clusters. **(B)** GO enrichment analyses of m6A-related DEGs.

Supplementary Figure 4 Characteristics of m6A score in TCGA. **(A)** Differences in m6A score between m6A clusters. **(B)** Differences in m6A score between m6A gene clusters. **(C)** Correlations between m6A score and tumor-infiltrating lymphocyte cells using Spearman analysis. **(D)** Forest plot of the alteration frequencies in two m6A score groups using chi-square test.
