# Supplementary material for: m6A regulator-based methylation modification patterns and characterization of tumor microenvironment in acute myeloid leukemia
Source: Front Genet. 2022 Aug 10;13:948079. doi: 10.3389/fgene.2022.948079 (PMC9399688; doi:10.3389/fgene.2022.948079)
Supplement: Supplementary file 1 [file DataSheet2.pdf]

Supplementary information

Figure S1. Characterization analysis of the 23 m6A regulators. (A) Principal component analysis 23 m6A regulators based on paired tumor-normal specimens. (B-C) Expression heat map of 23 m6A regulators in different types of immune cells (B) and hematological malignancies (C).

Figure S2. Unsupervised clustering of 23 m6A regulators in the TCGA cohort. (A-B) Consensus clustering analysis presented the relative change in the area under the cumulative distribution function (CDF) when k = 2–9. (C) The samples were clustered into cluster1, cluster2, and cluster3 when k = 3.

Figure S3. m6A phenotype-related DEGs in AML. (A) Venn diagram of 70 m6A-related DEGs among three m6A clusters. (B) GO enrichment analyses of m6A-related DEGs.

Figure S4. Characteristics of m6Ascore in TCGA. (A) Differences in m6A score between m6A clusters. (B) Differences in m6A score between m6A gene clusters. (C) Correlations between m6A score and tumor-infiltrating lymphocyte cells using Spearman analysis. (D) Forest plot of the alteration frequencies in two m6A score groups using chi-square test.
