# Supplementary figures and images for: m6A regulator-based methylation modification patterns and characterization of tumor microenvironment in acute myeloid leukemia
Source: Front Genet. 2022 Aug 10;13:948079. doi: 10.3389/fgene.2022.948079 (PMC9399688; doi:10.3389/fgene.2022.948079)

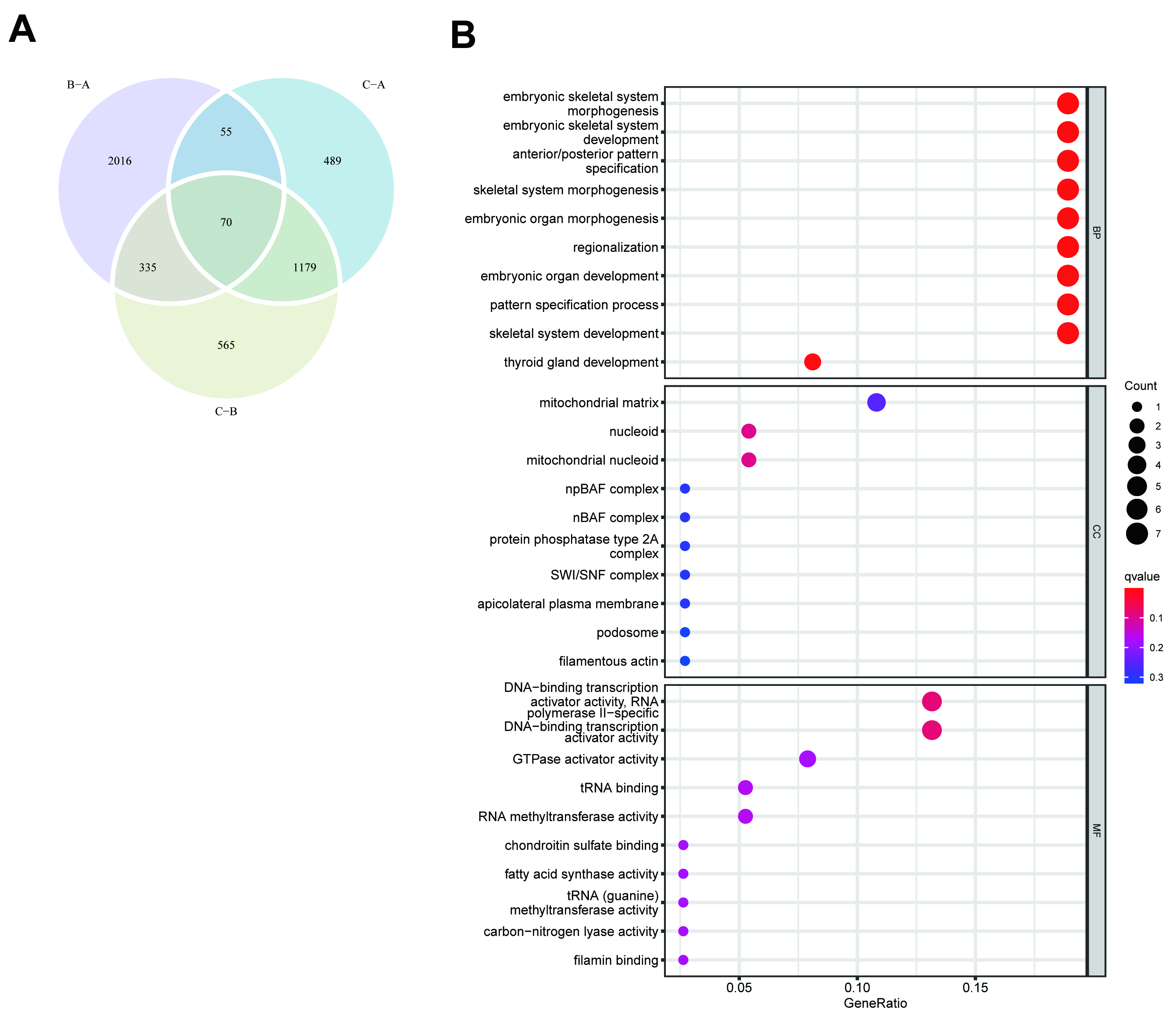

Supplement: Supplementary file 2 [file Image3.TIF]

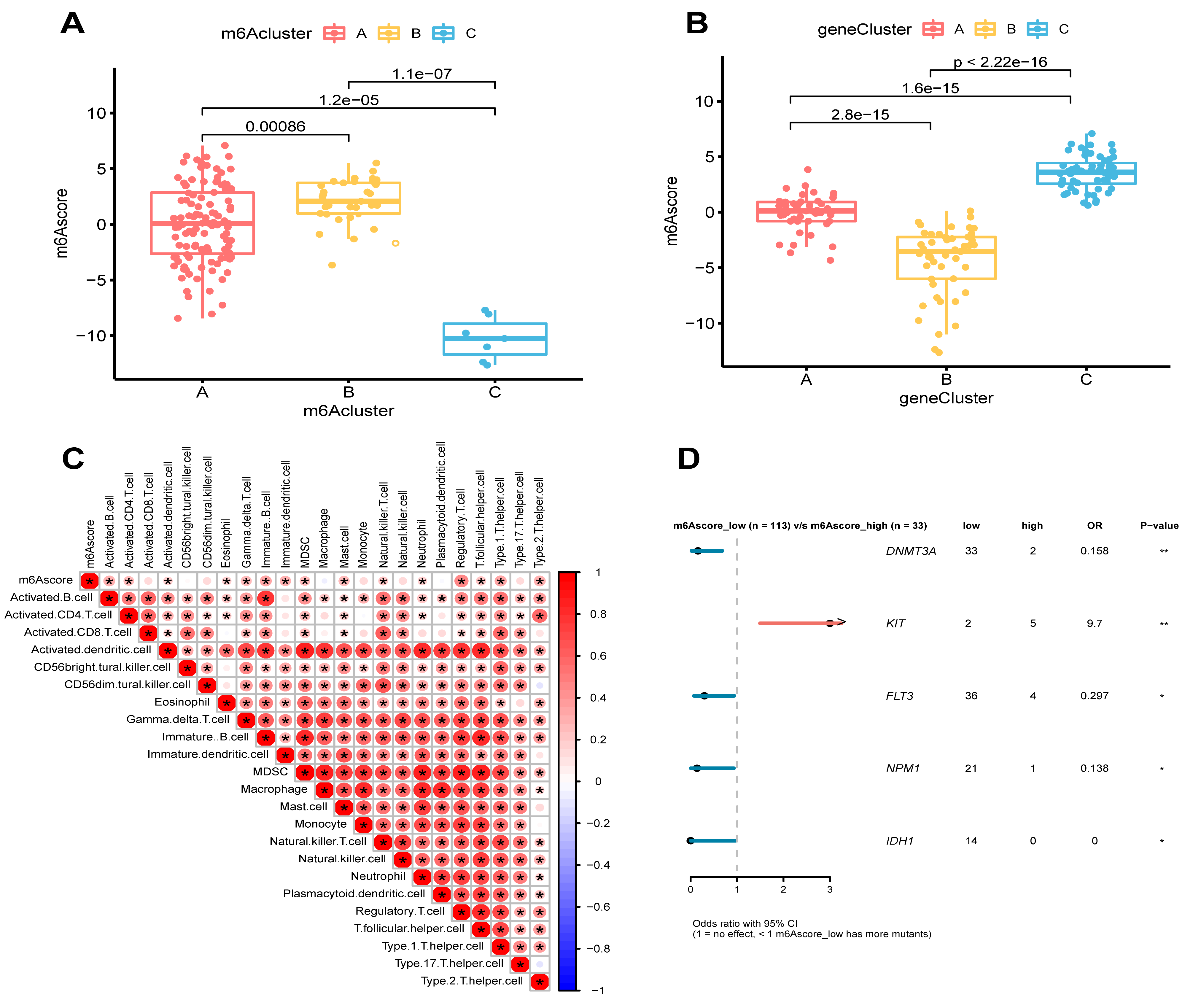

Supplement: Supplementary file 3 [file Image4.TIF]

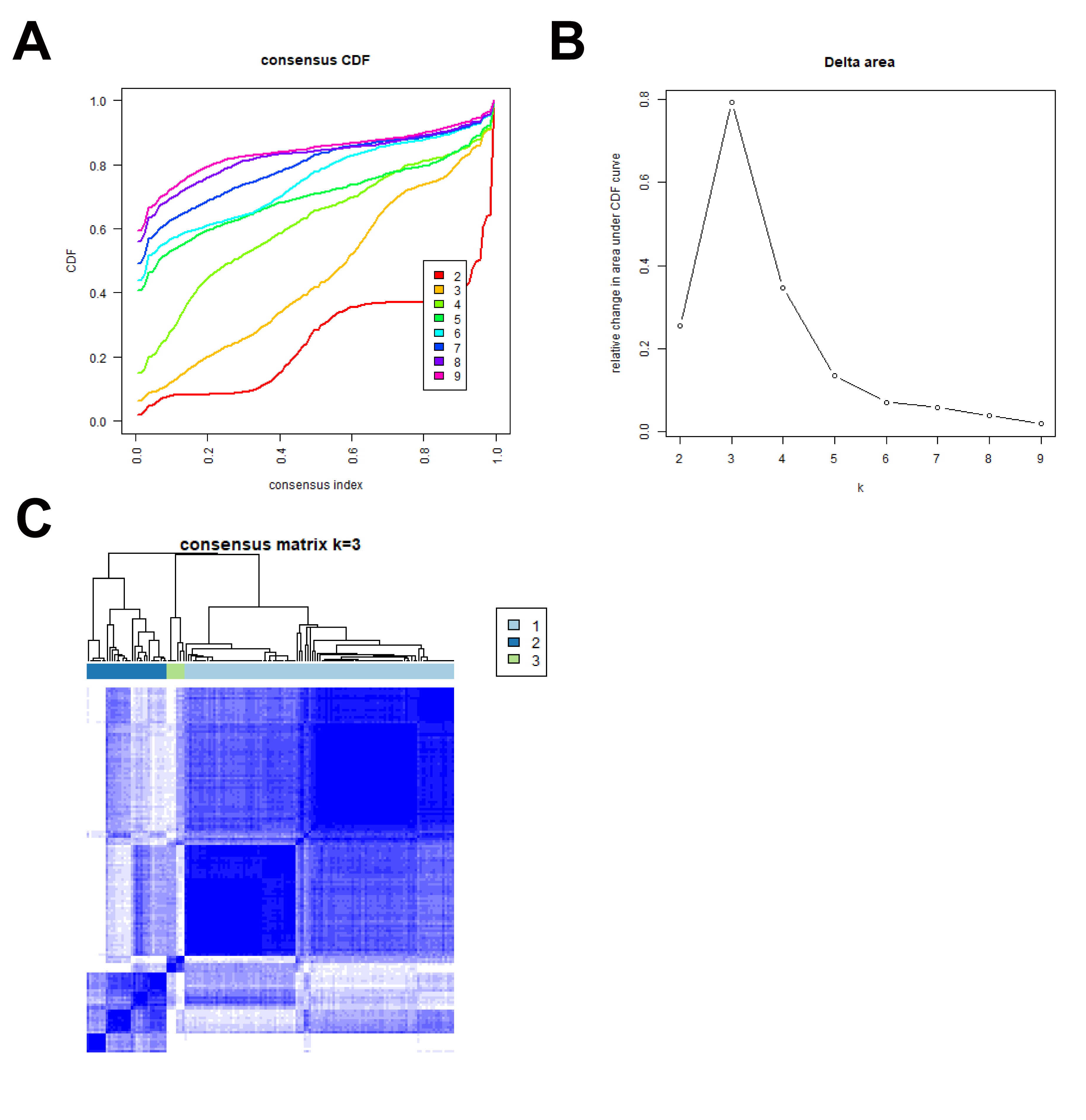

Supplement: Supplementary file 4 [file Image2.TIF]

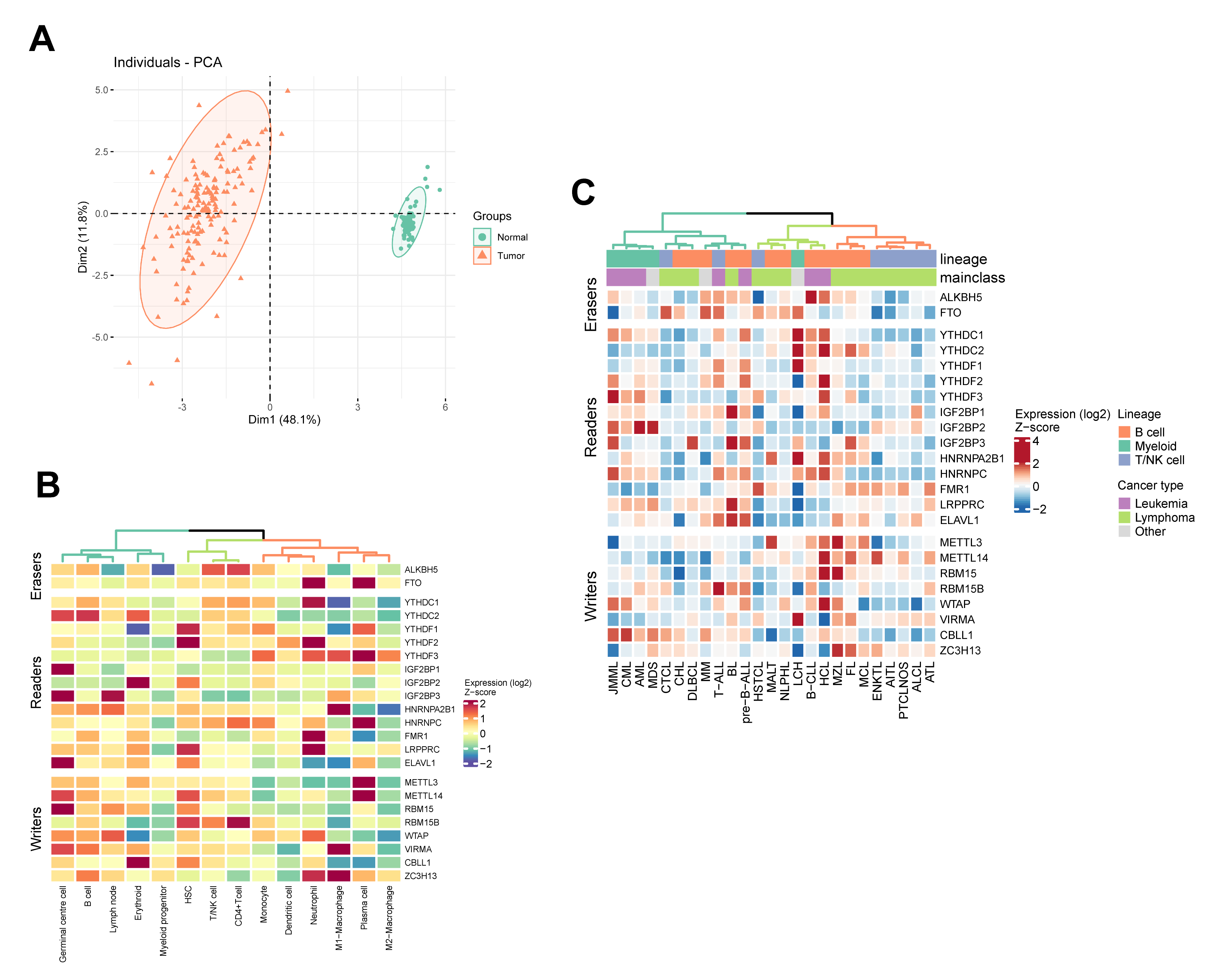

Supplement: Supplementary file 5 [file Image1.TIF]
